# PPARγ inhibitors enhance the efficacy of statin therapy for steroid-induced osteonecrosis of the femoral head by directly inhibiting apoptosis and indirectly modulating lipoprotein subfractions

**DOI:** 10.1371/journal.pone.0325190

**Published:** 2025-06-20

**Authors:** Kai-yun Chen, Xu-huan Li, Dan Chen, Shi-da Qian, Run-hong Mei, Qian Li, Xue-feng Yu, Xi-jing He

**Affiliations:** 1 Department of Orthopedic Surgery, Second Affiliated Hospital of Xi’an Jiaotong University, Xi’an, Shaanxi Province, China; 2 Affiliated Rehabilitation Hospital, Jiangxi Medical College, Nanchang University, Nanchang, Jiangxi, China; 3 First Affiliated Hospital of Hengyang Medical School, University of South China, Hengyang, Hunan, China; 4 Jiangxi Provincial Key Laboratory of Oral Diseases, Department of Stomatology, The First Affiliated Hospital, Jiangxi Medical College, Nanchang University, Nanchang, Jiangxi, China; 5 Orthopedic Hospital, Xi’an International Medical Center Hospital, Xi’an, Shaanxi Province, China; Duke University Medical Center: Duke University Hospital, UNITED STATES OF AMERICA

## Abstract

**Background:**

Steroid-induced osteonecrosis of the femoral head (SONFH) is a serious bone disease commonly seen in patients on long-term glucocorticoid therapy. Although statins have shown some efficacy in improving lipid metabolism, their efficacy in the treatment of SONFH remains limited. PPARγ inhibitors may enhance the efficacy of statins through several mechanisms. This study aims to investigate how PPARγ inhibitors may enhance the effects of statins in the treatment of SONFH by directly inhibiting apoptosis and indirectly modulating lipoprotein subfractions.

**Methods:**

We first treated osteoblasts in vitro with high concentrations of hormones to simulate the SONFH environment. We then treated the cells with either the PPARγ inhibitor GW9662, the statin lovastatin, or a combination of both. We assessed cell proliferation and apoptosis using CCK-8, flow cytometry and Western blotting. We then established a SONFH rabbit model using high doses of methylprednisolone and lipopolysaccharide. The rabbits were randomly divided into four groups: control group, lovastatin group, GW9662 group and combination therapy group. We observed hip joint MRI before treatment, after 4 weeks of treatment, and 4 weeks after stopping treatment. We performed hematoxylin-eosin staining of the femoral head and analysed serum lipoprotein subfractions using VAP technology. In addition, we used quantitative polymerase chain reaction (qPCR) to analyse the expression of genes related to lipid metabolism at week 3.

**Results:**

In vitro experiments showed that both GW9662 and lovastatin effectively inhibited hormone-induced apoptosis. In the animal studies, imaging and pathological results showed that the progression of SONFH was slower in the combination therapy group than in the other groups. VAP analysis showed that the lovastatin group had disturbed lipoprotein subfractions at the fourth week after stopping treatment, while the combination therapy group had more stable lipoprotein subfractions.

**Conclusion:**

PPARγ inhibitors significantly enhance the efficacy of statins in the treatment of SONFH by directly inhibiting apoptosis and indirectly modulating lipoprotein subfractions. These findings provide new insights into the clinical management of SONFH and suggest that combination therapy may be an effective strategy.

## 1. Introduction

Osteonecrosis of the femoral head (ONFH) is a refractory orthopaedic disease characterised by ischaemia and necrosis of bone cells, leading to trabecular bone destruction and clinical manifestations such as pain and collapse of the femoral head. Ultimately, this condition leads to joint dysfunction [1]. Long-term or excessive use of glucocorticosteroids (GCs) has been shown to significantly increase the incidence of steroid-induced ONFH (SONFH), despite the efficacy of GCs in the treatment of various conditions [2]. Notably, hormone therapy is associated with an increased risk of ONFH in global epidemics, such as coronavirus disease 2019 (COVID-19) and severe acute respiratory syndrome [3].

Currently, the progression of femoral head necrosis is rapid and the effectiveness of non-surgical treatments is limited, often requiring surgical intervention in most patients [4]. However, surgical treatment is associated with high costs and numerous potential complications. Therefore, there is an urgent need to identify more effective treatments beyond traditional regimens, such as anticoagulation, vasodilators, and lipid-lowering drugs.

While many studies have focused on SONFH and lipid metabolism in recent years, the relationship between SONFH and lipoprotein subfractions remains largely unexplored [5]. Previous research has shown that abnormalities in lipid metabolism, particularly elevated triglycerides (TGs) and low levels of high-density lipoprotein cholesterol (HDL-C), are closely associated with ONFH [6]. Blood lipid control alone cannot fully prevent ONFH, similar to the challenge of preventing atherosclerotic cardiovascular disease (ASCVD) – even with routine blood lipid management, the reduction in ASCVD incidence is less than 30% [7]. Consequently, there is a growing emphasis on clinical recommendations for lipid subfraction detection technologies, such as vertical auto profile (VAP), to predict diseases with serious sequelae [8].

Peroxisome proliferator-activated receptor gamma (PPARγ) is a nuclear receptor transcription factor primarily expressed in adipose tissue, liver, and muscle [9]. PPARγ plays a crucial role in regulating adipocyte differentiation, lipid metabolism, insulin sensitivity and the inflammatory response, making it important in the management of metabolic syndrome, type 2 diabetes and cardiovascular disease [10]. Previous studies have shown that long-term overuse of GCs can induce lipid abnormalities leading to steatosis and osteocyte necrosis. This process is thought to be an important pathogenic mechanism in the development of SONFH. Despite the well-documented role of PPARγ in various metabolic processes, its involvement in SONFH has not been thoroughly investigated. This research gap suggests that targeting PPARγ may offer new therapeutic strategies for SONFH.

## 2. Methods

### 2.1. Human osteoblast immortalized cells culture and identification

Human osteoblastic immortalised cells (OST) were obtained from Shanghai Saibokang Biotechnology Co., Ltd. These cells were cultured in a special medium for human osteoblastic immortalised cells (No. iCell-0099a-001b), also from the same company, in a constant temperature CO2 incubator (5% CO2, 37°C). Alkaline phosphatase (ALP) staining and alizarin red S (ARS) staining were used to identify and monitor the growth of mature human immortalised osteoblasts. The staining procedures were performed using an ALP staining kit (Beyotime, CHN) and an ARS staining kit (Beyotime, CHN) according to the instructions provided with the kits.

### 2.2. CCK-8 assay

A stock solution of methylprednisolone has been prepared and dissolved in DMSO to a concentration of 100mM. The stock solution was diluted to five different concentrations. The SIM tablet was dissolved in the medium to prepare the stock solution to a concentration of 100mM. Four different concentrations were obtained by gradient dilution. After seeding cells in 96 holes and waiting for the cells to attach to the bottom, different experimental groups were run separately. Each group is made up of at least 3 replicate wells. The main stock solution is diluted at least a thousand times to ensure that the final DMSO content is less than 0.1% (v/v). The treated cells are cultured for 48 hours in an incubator at 37°C and 5% CO2. After incubation, the medium was removed from each well and 90 µl of serum-free medium was added to each well. Add 10 µl of CCK-8 reagent per well and mix thoroughly. The 96-well plate is returned to the incubator and incubated for a further hour. After incubation, the optical density (OD) of each well is measured using an enzyme-linked immunoassay at a wavelength of 450 nm. The OD value reflects cell viability and proliferation ability and is an important index to evaluate the effect on cell growth. By comparing the OD values of the groups, suitable drug concentrations were screened for further experiments and the effects of the drugs on cell proliferation activity in the hormonal context were preliminarily evaluated.

### 2.3. OST cells were grouped and treated with drugs

After passage of the OST cells, drug intervention was performed by dividing the OST cells into five groups: control group, MP group (cells + 100 μM methylprednisolone), GW group (MP + 10 μM PPARγ inhibitor GW9662), ML group (MP + 10 μM lovastatin) and GL group (MP + 10 μM GW9662 + 10 μM lovastatin). Each group was treated for 48 h.

### 2.4. Flow cytometry

Apoptosis of OST cells was detected using Annexin V-FITC/PI apoptosis kit (Beyotime, CHN). Cells were harvested according to the instructions and resuspended several times. After mixing with the configured binding buffer in cold condition, the cells were detected by flow cytometer.

### 2.5. PPARγ and apoptosis-related proteins were detected by Western blotting

Cultured human osteoblasts were immortalised and grouped according to appropriate cell densities. Cells were harvested and lysed using protein lysis buffer to extract proteins. Cell debris was removed by centrifugation and the supernatant was collected. Protein concentration was determined using a protein assay method. The appropriate SDS-PAGE gel concentration was selected based on the size of the proteins. Equal amounts of protein were loaded onto the gel and separated by electrophoresis. The separated proteins were transferred from the gel to a PVDF membrane. Appropriate current and time were used to ensure complete transfer of the proteins. BSA was used to seal the membrane to prevent non-specific binding. The membrane was first incubated overnight at 4°C with primary antibodies against PPARγ (Proteintech, 16643–1-AP), caspase 9 (Proteintech, 10380–1-AP), cleaved-caspase 9 (Asp353) (Affinity, AF5240, 1250 RMB), BAX (Proteintech, 50599–2-Ig) and BCL-2 (Proteintech, 12789–1-AP). After washing, the membrane was incubated with HRP-conjugated secondary antibodies corresponding to the primary antibody species. Finally, the membrane was developed and exposed after staining with the developing solution.

### 2.6. Animal modeling and grouping

In this study, 60 adult New Zealand White rabbits (Sibeifu, CHN) weighing between 3.0 and 3.5 kg were selected. The rabbits were randomly divided into five groups: control group, model group, lovastatin treatment group, GW9662 treatment group and combined treatment group, with 12 rabbits in each group (with equal numbers of males and females).

Except for the control group, the remaining groups underwent an induction protocol to simulate disease conditions. The induction protocol included lipopolysaccharide (LPS, Sigma-Aldrich, USA) at a dose of 10 µg/kg, administered via the marginal ear vein every 24 hours for 2 days. Following LPS injection, methylprednisolone (MedChemExpress, USA) was administered at a dose of 20 mg/kg, injected into the right gluteal muscle every 24 hours for 3 consecutive days. Additionally, sodium penicillin (North China Pharmaceutical Co., Ltd., CHN) was administered at a dose of 80,000 U per injection into the left gluteal muscle, once every 24 hours for 7 days [4]. The control group received an equivalent volume of saline instead of LPS, methylprednisolone and sodium penicillin for the same duration of treatment.

In the lovastatin group, lovastatin was dissolved in physiological saline and administered by gavage at a dose of 100 µg/kg daily. In the GW9662 group, GW9662 was dissolved in physiological saline and administered by intraperitoneal injection at a dose of 5 mg/kg every 3 days (for a total of 10 injections over 30 days). The combined treatment group received 100 µg/kg lovastatin daily by gavage and 5 mg/kg GW9662 by intraperitoneal injection every 3 days. All treatments started 7 days after modelling and continued for 4 weeks.

During the administration process, all doses were calculated using the body surface area conversion formula for rabbits. Execution method: massive bloodletting after anesthesia for execution; Anesthesia method: Administer intravenous doses of 0.1 mg/kg metoclopramide, 3 mg/kg ketamine, and 0.3 mg/kg butorphanol. Relieve pain: Use anesthesia and analgesic drugs: Before experimental procedures that may cause animal pain, anesthesia drugs should be used reasonably according to the type, weight, and experimental requirements of the animal, so that the animal can accept the operation unconsciously. After surgery, appropriate analgesics should also be given as needed to alleviate the pain of the animals. Euthanasia: For experimental animals that are no longer needed, euthanasia should be used to ensure their painless death. The euthanasia method should comply with animal welfare requirements, such as using excessive anesthetics, carbon dioxide asphyxiation, and other fast and painless methods, and be operated by professionally trained personnel. Proper handling of animal carcasses: Experimental animal carcasses should be properly handled in accordance with relevant regulations, generally using harmless treatment methods such as incineration and deep burial to prevent disease transmission and environmental pollution. All procedures strictly adhered to the guidelines for the use of laboratory animals and were approved by the Medical Ethics Committee of the Affiliated Rehabilitation Hospital of Nanchang University.

### 2.7. Magnetic resonance imaging examination of rabbit hip joint

In the experiment, MRI examinations of both hip joints were performed on the experimental rabbits during the 3rd, 5th, and 9th weeks. The rabbits were under anesthesia, and scans were conducted using a PHILIPS Achieva 3.0T TX MRI scanner. The purpose of the scans was to observe soft tissue swelling around the bilateral hip joints, joint capsule effusion, joint space width, uniformity of the femoral head signal, as well as edema and collapse. This allowed for a comparison of imaging characteristics at 3 different points.

### 2.8. Lipoprotein subcomponent sample collection and VAP detection

Serum samples were collected at 2 and 3 weeks for the detection of VAP. At each time period, the serum of 5 rabbits from each group was randomly selected for the VAP test. The animals were fasted for 12 hours prior to sampling. A total of 2 ml of venous blood was collected and placed in a non-anticoagulant tube. After 1 hour at room temperature, the samples were centrifuged at 4000 rpm for 10 minutes at 4°C. The supernatant was collected, labelled and stored at −80°C.

During the analysis, thawed samples were mixed with potassium bromide and subjected to high speed centrifugation using a vertical rotor to obtain a density gradient. The centrifuge tubes were placed in a liquid handling device that allowed drainage from the bottom, with the flow controlled by a piston pump. The samples were centrifuged at a set speed, and the mixture was emptied while being mixed with the reagents. In a continuous flow, the samples and reagents were mixed, and the mixture was alternately aspirated through a Y-connector to draw in different liquids. The mixture was heated as it passed through a temperature controlled bath. When the reaction was complete, the samples were continuously fed into the flow cell of a spectrophotometer for absorbance measurement. The whole process was repeated three times, and the resulting data was subjected to statistical analysis. No data were missing or discarded at the end of the experiment.

### 2.9. HE stained the femoral head

After gross examination of the macroscopic structure, one side of the femoral head was placed in 4% paraformaldehyde for decalcification and subjected to HE staining. The specimen was fixed, decalcified, dehydrated, and embedded in paraffin. 5μm thick paraffin sections were prepared using a microtome. HE staining method was used, including steps such as dewaxing, staining, differentiation, counterstaining, and mounting. Observations focused mainly on changes in osteocytes and trabecular bone under light microscopy, as well as the amount of blood vessels and red blood cells in bone tissue, and changes in adipose tissue and eosinophil count. In addition, 10 random fields were selected under high magnification (400×) and 50 bone lacunae were counted in each field to calculate the rate of empty bone lacunae. The samples from the other side were used for the detection of lipid-related genes.

### 2.10. Ultrastructural transmission electron microscopy of femoral head

At week 5, thin sections of the rabbit femoral heads were fixed in 2% glutaraldehyde solution for 48 hours at 4°C. They were then rinsed three times with 0.1 mol/L-1 phosphate-buffered saline (PBS) for 15 minutes each time. They were then fixed in 2% osmium tetroxide for 2 hours and rinsed three times with PBS for 15 minutes each time. The samples were dehydrated in a graded series of ethanol: 50% → 70% → 80% → 90% → 100% → 100%, and finally in acetone, with each step taking 20 minutes. The samples were embedded in Epon812, and ultra-thin sections were cut using a diamond knife to a thickness of approximately 80um. Sections were stained with saturated uranyl acetate and lead citrate for 15 minutes each.

### 2.11. Lipid metabolism related genes of femoral head were detected by quantitative polymerase chain reaction

In the experiment, blood samples were taken from the experimental rabbits after intervention at weeks 2 and 3 to analyse the subfractions of lipoproteins. The rabbits were then euthanized, and the bilateral hindlimb femoral heads were collected and stored in a −80°C freezer for further use. The expression levels of genes related to lipid metabolism (Apolipoprotein (APO) A1, APOB, insulin-like growth factor binding protein-3 (IGFBP-3), PPARγ) were then measured.

To prepare the femoral head samples, bone fragments were obtained and ground into powder. TRIzol reagent was added to the powder for processing, followed by extraction of RNA extraction using chloroform, isopropanol, and ethanol. The concentration and purity of the extracted RNA was determined using an RNA quantification instrument. A reverse transcription reaction was performed, and the resulting cDNA was stored at −20°C (Supplementary S1 Table).

For qPCR, the PCR reaction system was prepared, and the PCR reaction conditions were set. The GAPDH gene was used as an internal reference, and the Ct values were recorded. The relative expression levels of each gene were calculated using the 2-ΔΔCt method. Each sample was assayed in triplicate to ensure the accuracy of the results.

### 2.12. Statistical methods and analysis software

When using SPSS 26.0 statistical software is used for statistical analysis, variables are usually presented as mean ± standard deviation. If the data meet the conditions of normal distribution and equal variance, comparisons between multiple groups are made using one-way analysis of variance (One-way ANOVA), and pairwise comparisons between groups are made using the LSD-t test. If the data do not follow a normal distribution, comparisons between multiple groups are made using a rank sum test, and specific pairwise comparisons are performed using the Kruskal-Wallis H test. A P-value of less than 0.05 is considered statistically significant. All experiments were repeated three times. Graphs were generated using GraphPad Prism 9 software, and Western blot analysis was performed using ImageJ software.

## 3. Results

### 3.1. Lovastatin plays an antagonistic role in hormone-induced apoptosis by silencing PPARγ

**Fig 1:**
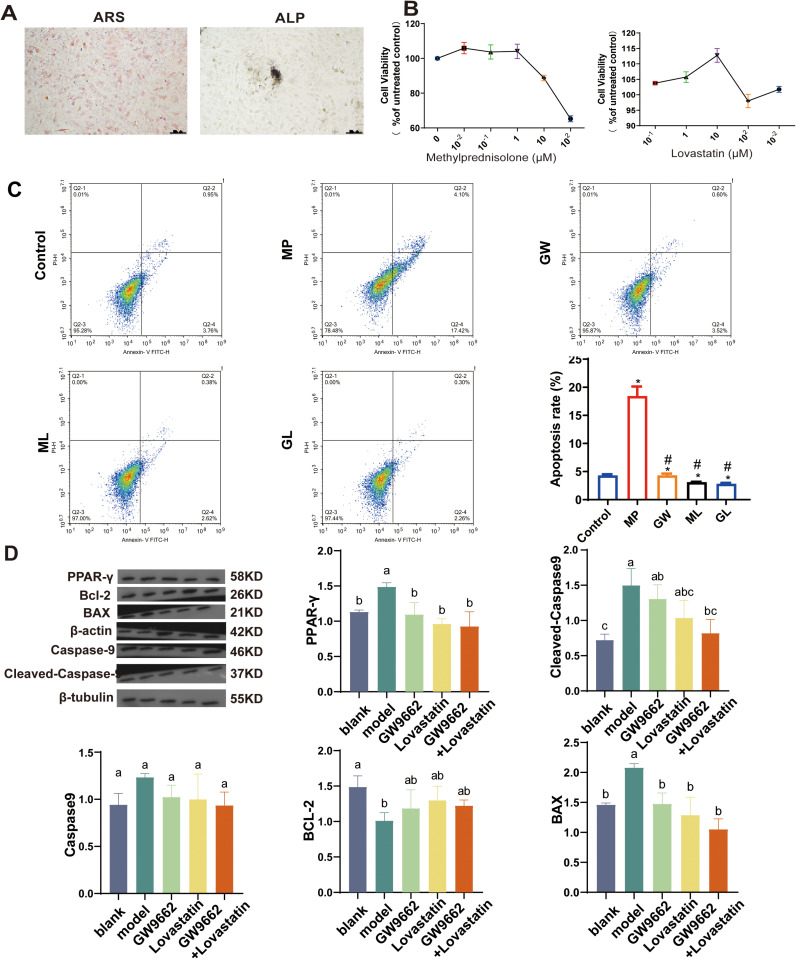
The experimental results of osteoblast – related research. (A). ARS and ALP- stained osteoblasts show brown-red color, indicating high mineralization with numerous, dense mineralized nodules, crucial for evaluating osteogenic differentiation. The cells have normal morphology with clear outlines and even cytoplasm. ALP – stained osteoblasts show strong activity, appearing dark blue – purple, reflecting high alkaline phosphatase expression during early osteogenic differentiation. (B). Cell proliferative activity after hormone and lovastatin treatment, revealing their impact on osteoblast growth. (C). The flow cytometry results showed the cell cycle and apoptosis status of cells in different treatment groups. (D). The WB detection results of PPAR – γ and apoptosis related protein expression changes elucidated the molecular mechanism.

OST cells typically enter the logarithmic growth phase after 3–5 generations of in vitro culture. This phase is characterised by active cell division, during which the number of cells increases rapidly (see Supplementary S1A Fig). The cells are spindle-shaped and adhere to the bottom of the culture dish. ALP staining is a commonly used method to assess osteoblast differentiation. ALP is an early marker of osteoblasts, and increased activity is usually associated with their differentiation and maturation. In ALP staining, the presence of grey-black granules in osteoblasts indicates ALP activity and confirms the osteogenic potential of the cells. ARS staining is another method of assessing the minerallising capacity of osteoblasts. This stain binds to calcium ions, causing calcium deposits in osteoblasts to appear red. A positive ARS staining result indicates that mineralisation has occurred in OST cells during culture, which is a key feature of mature osteoblasts (Fig 1A).

Methylprednisolone, GW9662, and lovastatin were administered to immortalised human osteoblasts at different concentrations for 48 hours. Qsteoblasts proliferation was assessed using the CCK-8 assay (see Fig 1B). At a concentration of 100 μM, methylprednisolone induced a proliferation rate of approximately 50% in osteoblasts. However, at 1000 μM, the proliferation rate decreased significantly. This suggests that high concentrations inhibit osteoblast proliferation excessively, which is detrimental to subsequent experiments. Therefore, 100 μM was chosen as the intervention dose for methylprednisolone. Both GW9662 and lovastatin showed the most significant promoting effects on osteoblast proliferation at a concentration of 10 μM. These concentrations were therefore selected for subsequent experimental interventions.

Evaluation of the proliferation activity of cells in each group revealed that GW9662 and lovastatin exerted protective effects under hormonal conditions (Fig 1B). After 48 hours of different experimental treatments, ARS staining showed calcium deposition in OST cells. This indicates that OST cells maintain their osteogenic activity under different conditions. This stable osteogenic activity is important for bone tissue engineering research and the treatment of bone diseases (see Supplementary in S1B Fig, Supplementary S2–S4 Figs).

CCK-8 assay results showed no significant difference in the effects of GW9662 and lovastatin on osteoblasts. Flow cytometry showed that hormones can induce cell apoptosis, but all treatment groups were able to inhibit hormone-induced apoptosis. Furthermore, the PPARγ inhibitor did not significantly enhance the anti-apoptotic function of lovastatin (Fig 1C).

To investigate whether the PPARγ pathway is involved in the antagonistic effect of lovastatin on hormone-induced cell apoptosis, Western blotting was used to detect the expression changes of PPARγ and apoptosis-related proteins in each experimental group. After hormone treatment, PPARγ expression was upregulated, while Bcl-2 levels decreased. In contrast, the pro-apoptotic proteins Bax and Cleaved-Caspase 9 showed increased expression. However, after treatment with lovastatin and GW9662 alone, both PPARγ and Bcl-2 expressions increased. There was no significant difference between the combined treatment group and the individual treatment groups, indicating that PPARγ is an important upstream factor through which lovastatin directly antagonizes hormone-induced cell apoptosis (Fig 1D).

### 3.2. Lovastatin combined with PPARγ inhibitors may be more effective in treating SONFH

**Fig 2:**
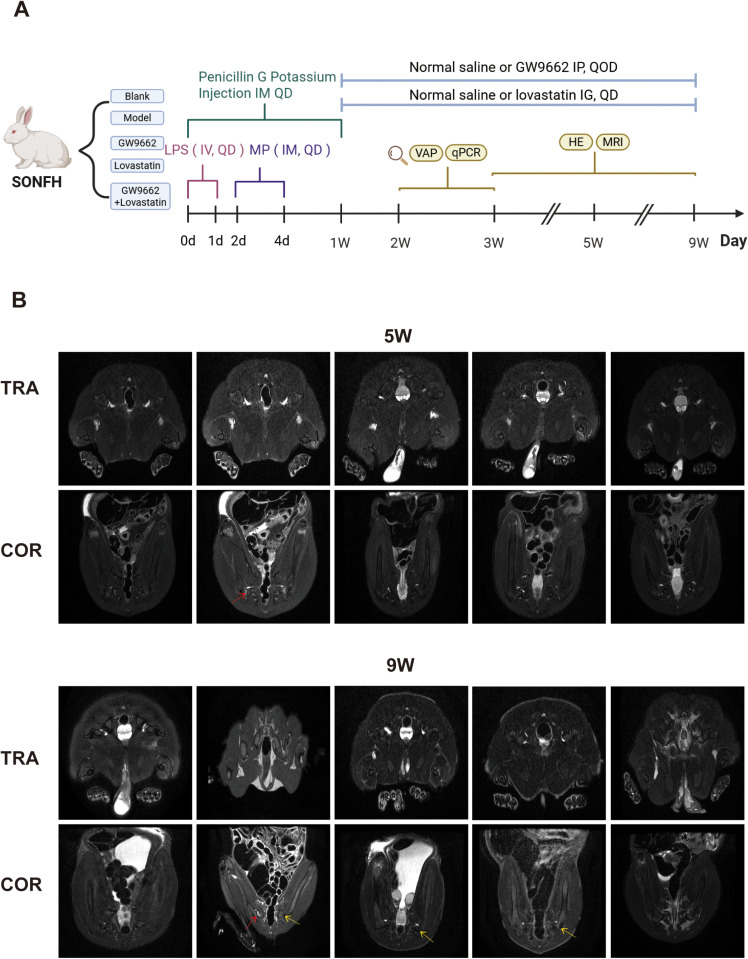
Flow chart of animal experiment section (A) and hip T2WI results were detected by MRI (from left to right:Blank, Model, GW9662, Lovastain, GW9662 + Lovastain) (B). Red arrows indicate changes in the bone marrow edema signal, and yellow arrows indicate changes in ischemia.

MRI is currently the most widely used diagnostic tool in clinical practice to assess the onset and progression of SONFH. It is an essential method for the early diagnosis of femoral head necrosis. To confirm the successful establishment of the SONFH model and the progression of the disease, MRI scans of the hip joints of the experimental rabbits were performed at 3, 5, and 9 weeks (Fig 2B). At week 3, the model group showed no significant abnormalities on T2-weighted imaging (T2WI), and there was no statistically significant difference compared to the control group. However, at week 5, the model group showed irregular patchy high signals in the bilateral joint capsules on T2WI, together with a slight widening of the hip joint space. These findings are consistent with the early imaging characteristics of ONFH. At week 9, there was cortical discontinuity, a blurred growth plate, narrowed hip joint space, irregular high signals in the subchondral bone marrow of the femoral head, flattening of the femoral head, and black ischaemic lesions. The control group showed no abnormalities at any time point, indicating the successful establishment of the progressive SONFH model. At week 3, no significant abnormalities were observed in any of the treatment groups. At week 5, all treatment groups showed signs of bone marrow oedema and an increased joint space, but the femoral heads remained smooth with no apparent low signals. Notably, the GW9662 treatment group had a larger area of high signal compared to the other treatment groups. At week 9, the GW9662 group showed bilateral joint capsule effusion, narrowed hip joint space, localised ischaemic lesions, and partial deformity of the femoral head. In contrast, the combined treatment group showed no significant dark areas and maintained a round appearance. The lovastatin group showed mild widening of the bilateral hip joint space bilaterally, with scattered high signals near the proximal femur, but no obvious deformity of the femoral head was observed.

### 3.3. GW9662 can be used in combination with statins to stabilize the disturbed lipoprotein subcomponents more quickly

We discussed the differences in lipoprotein subfractions at both time points to further characterise the potential relationship between SONFH and lipoprotein subfractions. At week 2, TG, total cholesterol (TC), and high-density lipoprotein (HDL) levels were higher in the model group than in the blank group (*P = *0.0043, *P* = 0.0022, *P* = 0.0053). The TG level in the combined group was significantly lower than that in the model group (*P = *0.0181). Low -density lipoprotein (LDL) levels in the GW9662 group were lower than that in the model group (*P* = 0.0324). HDL levels were lower in the model, GW9662 and lovastatin groups were lower than those in the blank group (*P = *0.0053, *P* = 0.0058, *P* = 0.0080). At week 3, TG levels remained higher in the model group than in the sham group (*P = *0.0069), and TG levels in the lovastatin and the combination groups were lower than in the model group (*P = *0.0163, *P* = 0.0054) (Table 1).

**Table 1:** Data of TG, TC, LDL and HDL of each group in week 2 and 3 (X̄ ± S, mmol/L).

Group		Blank	Model	GW9662	lovastatin	Combined
2W	TG	0.82 ± 0.16^#^	20.61 ± 9.52^*^	19.60 ± 11.05	13.24 ± 3.97	3.91 ± 2.61^#^
	TC	1.65 ± 0.24^#^	6.78 ± 3.27^*^	3.79 ± 1.56	2.64 ± 0.69	2.40 ± 0.28
	LDL-C	0.43 ± 0.20	1.84 ± 1.57	0.34 ± 0.20^#^	0.48 ± 0.31	0.61 ± 0.35
	HDL-C	0.92 ± 0.11^#^	0.36 ± 0.20^*^	0.36 ± 0.05^*^	0.34 ± 0.16^*^	0.87 ± 0.36
3W	TG	0.96 ± 0.14^#^	2.55 ± 0.79^*^	2.13 ± 0.97	1.12 ± 0.13^#^	0.92 ± 0.20^#^
	TC	1.59 ± 0.39	3.29 ± 1.50	1.41 ± 0.61	1.28 ± 0.18	1.56 ± 0.41
	LDL-C	0.37 ± 0.13	1.71 ± 1.19	0.46 ± 0.34	0.26 ± 0.08	0.35 ± 0.16
	HDL-C	1.1 ± 0.18	0.73 ± 0.20	0.53 ± 0.22^*^	0.80 ± 0.18	0.92 ± 0.30

Note: *indicates P < 0.05 compared with the blank group and # indicates P < 0.05 compared with the model group.

**Fig 3:**
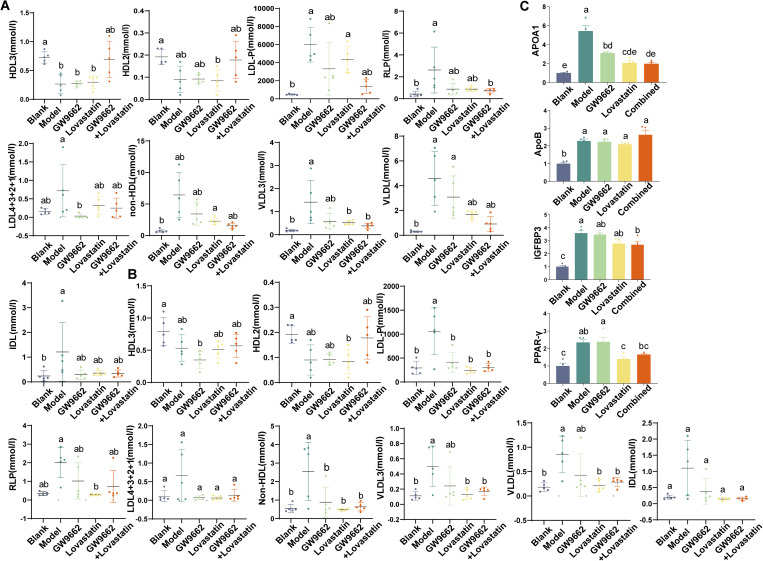
Lipoprotein subfraction plots for each experimental group in week two(A). Lipoprotein subfraction plots for each experimental group in week three(B).Detection of gene expression levels in femoral head tissues of experimental groups by qPCR method(C).

HDL_2_ and HDL_3_ levels in the model group were lower than those in the sham group at both time points (*P* < 0.05). At week 2, HDL_3_ levels were significantly lower in the model, lovastatin and GW9662 groups than those in the sham group (*P* = 0.0034, *P* = 0.0066, *P* = 0.0037). HDL_2_ levels in the lovastatin group were lower than that in the sham group (*P* = 0.0381). LDL-P levels in the model and lovastatin groups were significantly higher than those in the blank group (*P* = 0.0013, *P* = 0.0127). Remnant lipoprotein (RLP) levels were lower in the combination and placebo groups than in the model group (*P = *0.0434, *P* = 0.0160). LDL_4+3+2+1_ levels in the GW9662 group were lower than those in the model group (*P = *0.0475). The level of non-HDL was higher in lovastatin group than in the sham group (*P* = 0.0038). Very low density lipoprotein 3 (VLDL_3_) levels were lower in the sham, lovastatin and combination groups were lower than those in the model group (*P = *0.0030, *P* = 0.0390, *P* = 0.0136). The VLDL level in the lovastatin group was higher than that in the sham group (*P = *0.0015) (Fig 3A, B).

At week 3, HDL3 levels were lower in the GW9662 group was lower than in the sham group (*P* = 0.0068). HDL2 levels in the model and GW9662 groups were lower than in the sham group (*P = *0.0482, *P* = 0.0394). LDL-P levels in the sham, GW9662, lovastatin and combined groups were lower than those in the model group (*P =* 0.0008, *P* = 0.0043, *P* = 0.0004, *P* = 0.0009). RLP levels in the lovastatin group were lower than those in the model group (*P = *0.0127). Levels of non-HDL were lower in the sham, GW9662, lovastatin and combination groups than those in the model group (*P =* 0.0063, *P* = 0.0267, *P* = 0.0045, *P* = 0.0091). VLDL3 levels in the sham, lovastatin and combination groups were lower than those in the model group (*P =* 0.0139, *P* = 0.0208, *P* = 0.0439). VLDL levels in the sham, lovastatin and combination groups were lower than those in the model group (*P* = 0.0061, *P* = 0.0096, *P* = 0.0236).

When measuring Lp(a) value by immunoturbidimetry, the use of human antibodies in the rabbit model has been controversial. In addition, due to the polymorphism of Apo(a), the results of different Lp(a) assays are not entirely consistent, and the results are reported in units of nmol/L and mg/L, which cannot be directly converted. Therefore, we believe that calculated Lp(a) value calculated is more reliable. Lp(a) levels were similar in each group at both time points, and a more sensitive method may be needed to detect Lp(a).

qPCR was used to determine the mRNA expression levels of APOA1, ApoB, IGFBP3, and PPARγ genes in the femoral head bone tissue of the different experimental groups. However, at week 3, the mRNA expression levels of these four genes were significantly higher in the model group than in the control group. After long-term treatment, all treatment groups effectively inhibited the activity of these lipid metabolism-related genes. Notably, at week 3, the combined treatment group showed a more sustained reduction in PPARγ gene activity compared to lovastatin alone (Fig 3C).

### 3.4. GW9662 can assist statin therapy in the treatment of SONFH

**Fig 4:**
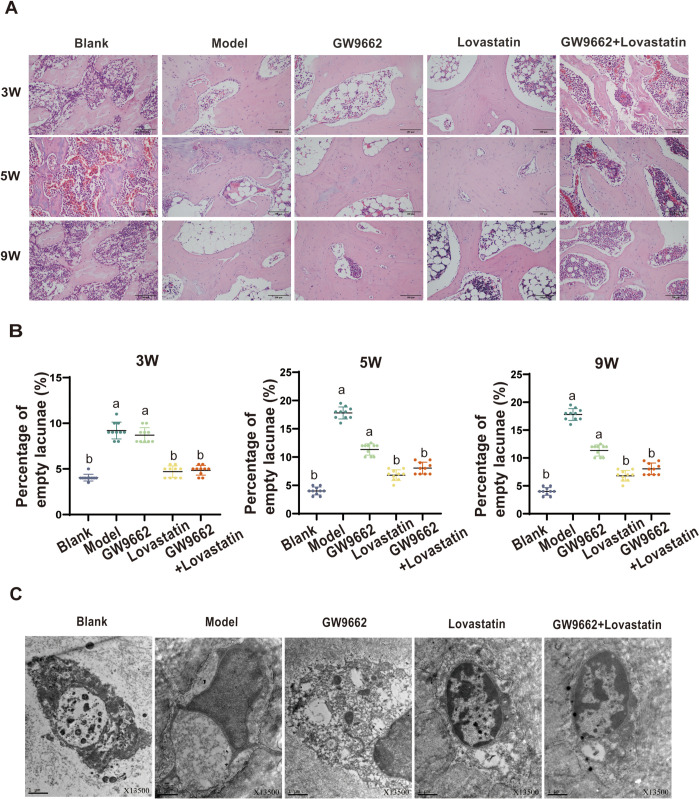
HE staining was performed on the femoral head tissue after blood collection (A), and the rate of empty lacunae was calculated (B). Additionally, the ultrastructure of the cells was observed by transmission electron microscopy (C).

We compared the structural and histological characteristics of the bone in the different groups, as well as the changes in the void to bone ratio at 3, 5, and 9 weeks (Fig 4A, B). Throughout the study, the control group maintained good trabecular continuity and structure. Osteocytes were dense and well nourished, indicating adequate blood supply and healthy bone tissue growth. In the model group, the trabecular bone became progressively thinned with some fractures and structural disarray. There was an increase in empty bone spaces, and the number and volume of bone marrow adipocytes gradually increased, indicating degeneration and damage to the bone tissue. In the combined treatment group at 3, 5, and 9 weeks, the trabecular structure showed good continuity, fewer empty bone spaces and a dense osteocyte structure. In particular, the number of adipocytes was significantly lower than in the model group. The lovastatin group also showed continuous trabecular structure and dense osteocytes, with fewer adipocytes at 3 and 5 weeks. However, by week 9, the trabecular bone had thinned and there was an increase in trabecular voids, eosinophils, and adipocytes, indicating that the protective effect of lovastatin may diminish over time. After 3 weeks, the cavity ratio was significantly higher in the model and GW9662 groups than in the control group. However, the combined treatment and lovastatin groups had lower empty cavity ratios compared to the model group, which were not significant different from the control group. At 5 and 9 weeks, the model group had a significantly higher empty cavity ratio than the control group, with no statistically significant difference when compared to the GW9662 group. At 5 weeks, there was no significant difference in the empty cavity ratio between the combination treatment and lovastatin groups, but at 9 weeks, the empty cavity ratio in the lovastatin group was lower than in the model group.

Ultrastructural observations of the femoral head in the sham, model, and treatment groups at 4 weeks showed significant differences. In the blank group, abundant organelles were visible in the osteocytes, with particularly prominent rough endoplasmic reticulum and no obvious organelle abnormalities. The model group showed clear signs of osteocyte necrosis characterised by reduced organelles, swollen mitochondria, structural disoreder, shortened or absent cristae, reduced lipid droplets, wrinkled nuclear membranes, and cytoplasmic condensation.

An increase in organelles, including mitochondria, was observed in the lovastain and combination treatment groups. Swelling was reduced, and the organelles appeared more oval or rod-shaped, with clear cristae and membrane structures. Lipid droplets were significantly reduced (Fig 4C).

## 4. Discussion

In this study, we investigated the potential mechanisms of lovastatin combined with PPARγ inhibitors in the treatment of SONFH, with a particular focus on the importance and novelty of regulating lipoprotein subfractions in this treatment approach. Lovastatin, a commonly used lipid-lowering drug, has recently been showed to have significant anti-apoptotic effects [11]. PPARγ plays an important role in bone metabolism and is mainly expressed in adipocytes and osteoblasts and is involved in the regulation of adipocyte differentiation and bone metabolism [12]. Lovastatin enhances its anti-apoptotic effects by suppressing PPARγ [11]. The synergistic effect of lovastatin and PPARγ inhibitors is a typical example of how combination therapy can improve treatment outcomes through complementary mechanisms.

Lovastatin enhances its anti-apoptotic effects by silencing PPARγ. Typically, activation of PPARγ is associated with the inhibition of apoptosis, while lovastatin suppresses PPARγ expression through specific signalling pathways, such as MAPK (Mitogen-Activated Protein Kinase) and PI3K/Akt pathways, thereby indirectly promoting cell apoptosis [13]. This mechanism suggests that lovastatin may have potential applications in the treatment of diseases associated with cell apoptosis. The protective effects of Lovastatin on bone cells are mainly achieved by regulating bone metabolism and inhibiting osteocyte apoptosis. Osteocytes are one of the main cell types in bone tissue and their dysfunction can lead to diseases such as osteoporosis [14]. By inhibiting the expression of PPARγ, lovastatin reduces the apoptosis of bone cells, thereby preserving their normal function. In addition, lovastatin can regulate the expression of bone metabolism-related genes, promoting bone formation and inhibiting bone resorption, further enhancing the protective effects on bone cells [15]. These mechanisms suggest that lovastatin may have broad application prospects in bone protection.

Combination therapy is playing an increasingly important role in modern medicine, especially in the management of complex diseases [16]. The synergistic effect of lovastatin and PPARγ inhibitors is a typical example of how combination therapy can improve outcomes through complementary mechanisms. Lovastatin mainly lowers cholesterol levels by inhibiting HMG-CoA reductase, while PPARγ inhibitors are mainly used to regulate glucose and lipid metabolism [17]. When used together, they can effectively control lipid levels and improve insulin resistance, leading to better metabolic control.

The use of combination therapy is particularly important in the treatment of complex diseases. For example, in the treatment of diabetes with cardiovascular disease, a single drug is often not sufficient to control the disease. Combining antihyperglycaemic and antihyperglycaemic agents with cardiovascular agents can help contorl blood glucose levels more effectively and reduce the incidence of cardiovascular events [18]

The management of SONFH demonstrates the unique value of combination therapy. SONFH is a common orthopedic condition that often requires a comprehensive approach. The combined use of anti-inflammatory drugs, vasodilators, and bone metabolism regulators together can effectively reduce inflammation, improve local blood flow and promote repair and regeneration of bone tissue [19].

Lipoprotein subfractions play a crucial role in lipid metabolism. Lipoproteins are the primary carriers of lipids in the blood, including cholesterol, triglycerides, and phospholipids. Different types of lipoproteins [20], such as LDL and HDL, have specific functions in lipid metabolism. LDL primarily transports cholesterol from the liver to peripheral tissues, whereas HDL transports cholesterol from peripheral tissues back to the liver for metabolism and excretion [21]. Abnormal distribution and dysfunction of lipoprotein subfractions are closely associated with several cardiovascular diseases, including atherosclerosis and coronary heart disease [22].

The effect of steroid treatment on lipoprotein subfractions is considerable. Steroid drugs, such as glucocorticoids, are commonly used to treat various inflammatory and autoimmune diseases [23]. However, long-term use of these drugs can disrupt lipid metabolism, resulting mainly in increased LDL levels and decreased HDL levels [24]. This alteration in lipoprotein subfractions increases the risk of cardiovascular disease in patients. In addition, steroid treatment may also result in increased triglyceride levels, exacerbating lipid metabolism disorders [25]. Therefore, it is important to monitor and regulate lipoprotein subfractions during steroid treatment.

The regulation of lipoprotein subfractions has potential implications for the treatment of SONFH. The pathogenesis of SONFH is closely linked to dyslipdaemia. Studies show that abnormal distribution and dysfunction of lipoprotein subfractions may lead to the development of SONFH. By regulating lipoprotein subfractions, such as lowering LDL levels and increasing HDL levels, it may be possible to improve the lipid metabolic status of SONFH patients and thereby slow disease progression [26]. In addition, drug treatments and lifestyle interventions targeting lipoprotein subfractions may become new strategies for the treatment of SONFH.

The dual mechanism of lovastatin and PPARγ inhibitors offers new hope for the treatment of SONFH. Lovastatin reduces cholesterol synthesis by inhibiting HMG-CoA reductase, thereby reducing lipid accumulation in the bone marrow. PPARγ inhibitors, on the other hand, reduce adipocyte differentiation by inhibiting PPARγ activity, thereby improving the fat content in the bone marrow [27]. The combined use of these two drugs can have a synergistic effect, effectively improving the symptoms of SONFH. Studies have shown that this dual-mechanism treatment strategy has shown good results in clinical trials, providing a new option for the treatment of SONFH.

Innovative treatment approaches have broad potential for use in the management of SONFH. As research into the pathogenesis of SONFH deepens, more innovative treatment ideas are being proposed. For example, gene therapy, stem cell therapy, and the use of biomaterials all offer new possibilities for treating SONFH. Gene therapy aims to repair or replace defective genes, thereby addressing the underlying cause of SONFH [28]. Stem cell therapy promotes bone marrow regeneration and repair by transplanting healthy stem cells [29]. The use of biomaterials can provide a favorable microenvironment for the bone marrow and promote normal osteocyte function. The implementation of these innovative treatment strategies could revolutionise the management of SONFH and improve the quality of life of patients.

The clinical application of lipoprotein subfraction regulation is an emerging field with significant potential. Lipoprotein subfractions, such as LDL and HDL, have important implications for assessing cardiovascular disease risk assessment and treatment. Research indicates that by regulating the ratio and structure of lipoprotein subfractions can effectively reduce the incidence of cardiovascular diseases. For example, the anti-inflammatory and antioxidant functions of HDL can be enhanced by drug interventions or lifestyle changes, thereby slowing the progression of atherosclerosis [30]. In addition, regulation of lipoprotein subfractions can be used to assess patient response to treatment and prognosis, providing a basis for personalized treatment.

The use of biomarkers related to lipid metabolism is of great value in clinical diagnosis and management. These biomarkers include cholesterol, triglycerides, and lipoprotein(a), which can reflect an individual’s lipid metabolism status and risk of cardiovascular disease. By measuring the levels of these biomarkers, doctors can identify high-risk patients early and take appropriate preventive measures. For example, high levels of lipoprotein(a) are strongly associated with an increased risk of atherosclerosis and cardiovascular events, making the monitoring and regulation of lipoprotein(a) levels an important strategy for the prevention and treatment of cardiovascular diseases [31]. In addition, dynamic monitoring of biomarkers related to lipid metabolism can be used to assess treatment efficacy and adjust treatment plans.

The development of personalised treatment plans is an important direction in modern medicine, particularly in the prevention and treatment of lipid metabolism and cardiovascular disease. The development of personalised treatment plans requires a comprehensive consideration of various factors, including a patient’s genetic background, lifestyle, metabolic status, and clinical presentation. Through genetic testing and biomarker analysis, it is possible to identify patient groups that respond well to certain drugs or treatment strategies, enabling precision medicine [32]. For example, some patients may respond poorly to certain lipid-lowering drugs, and personalised treatment plans can help select more appropriate drugs or combination therapies to improve outcomes and quality of life. In addition, personalised treatment plans can be regularly monitored and adjusted to ensure safety and effectiveness.

In conclusion, this review thoroughly discusses the potential mechanisms of action of lovastatin combined with PPARγ inhibitors in the treatment of SONFH, particularly in regulating lipoprotein subfractions, improving lipid metabolism, and reducing osteocyte apoptosis. These findings not only provide a new theoretical basis for the molecular treatment of SONFH but also suggest potential improvements in clinical treatment strategies. Although current research results shows significant effects of lovastatin combined with PPARγ inhibitors in the treatment of SONFH, caution is warranted. Firstly, the sample sizes are relatively small, and most of the studies are animal studies without the support of large clinical trials. Therefore, these preliminary findings need to be validated in larger clinical trials to ensure their safety and efficacy.

Secondly, there are certain discrepancies between different studies, which may be related to experimental design, sample selection, and drug dosage. Therefore, future research should focus on standardising experimental designs and ensuring data transparency to guarantee the reproducibility and reliability of results. Furthermore, given the complexity and multifactorial nature of SONFH, a single drug or combination therapy may not fully address all issues. Therefore, future studies should explore multi-target and multi-pathway comprehensive treatment strategies to achieve better therapeutic outcomes.

## 5. Conclusion

In conclusion, the preliminary research results on lovastatin combined with PPARγ inhibitors in the treatment of SONFH are encouraging, but further in-depth research and clinical validation are needed. By balancing different research perspectives and findings, we can better understand the potential value and limitations of this treatment strategy and ultimately provide more effective and safer options for SONFH patients.

## Supporting information

S1 Table (DOCX)

S1.Fig (RAR)

S2 Fig (RAR)

S3 Fig (RAR)

S4 Fig (RAR)

## Abbreviations

SONFH: Steroid-induced osteonecrosis of the femoral head
ONFH: osteonecrosis of the femoral head
PCR: Polymerase chain reaction
qPCR: quantitative polymerase chain reaction
VAP: vertical auto profile
GCs: glucocorticoids
TG: triglycerides
TC: total cholesterol
HDL: high density lipoprotein
LDL: low density lipoprotein
RLP: Remnant lipoprotein
VLDL: very low density lipoprotein
HDL-C: high-density lipoprotein cholesterol
ASCVD: atherosclerotic cardiovascular disease
OST: osteoblastic immortalized cells
ALP: alkaline phosphatase
ARS: alizarin red-S
OD: optical density
LPS: lipopolysaccharide
PBS: phosphate-buffered saline
HE: Hematoxylin-eosin.
