# Supplementary material for: PPARγ inhibitors enhance the efficacy of statin therapy for steroid-induced osteonecrosis of the femoral head by directly inhibiting apoptosis and indirectly modulating lipoprotein subfractions
Source: PLoS One. 2025 Jun 20;20(6):e0325190. doi: 10.1371/journal.pone.0325190 (PMC12180619; doi:10.1371/journal.pone.0325190)
Supplement: S1 Table — (DOCX) [file pone.0325190.s001.DOCX]

**Supplemental Table1** Primers for quantitative real-time PCR

| Gene | 5’ forward primer | 3’ reverse primer |
| --- | --- | --- |
| GAPDH | CATCAAGAAGGTGGTGAAGCA | AGCATCGAAGGTAGAGGAGTG |
| APOA1 | TTTCGCCACCGTGTATGTGG | CAGGAGCTTCAGGTTGAGTTG |
| APOB | AATGTCATCAACACAGTCCAAGA | ACTCTCCAGCCAACTACACTT |
| IGFBP3 | TGTCCGATGGTTGATCTCTCGTGCTA | GGTTCGATCTGATGGATGTCTCTT |
| PPAR-γ | CAAGGCTTCATGACGAGGGA | ATGTCCTCGATGGGCTTCAC |
